# Preventing calcium and vitamin D deficiencies following weight loss and metabolic surgery

**DOI:** 10.1186/s12893-021-01348-3

**Published:** 2021-09-25

**Authors:** Reshi Suthakaran, Imeshi Indigahawela, Krinal Mori, Kiat Lim, Ahmad Aly

**Affiliations:** 1 Austin Hospital, Heidelberg, VIC Australia; 2 The Northern Hospital, Epping, VIC Australia

**Keywords:** Gastric bypass, Sleeve gastrectomy, Calcium, Vitamin D, Parathyroid hormone

## Abstract

**Introduction:**

Uncertain nutritional outcomes following common metabolic surgical techniques are concerning given the long-term potential for postoperative metabolic bone disease. This study aims to investigate the variations in serum calcium, vitamin D, and parathyroid hormone (PTH) levels following Roux-en-Y Gastric bypass (RYBP) and Sleeve Gastrectomy (SG).

**Methods:**

A retrospective analysis of 370 patients who underwent metabolic surgery at a single-centre group practice in Melbourne, Australia, over 2 years.

**Results:**

Patients underwent SG (n = 281) or RYGP (n = 89), with 75% and 87% of the cohort being female, respectively. Postoperative mean serum calcium levels and median serum vitamin D levels improved significantly by 24 months within both cohorts. Serum PTH levels within the RYBP group were significantly higher than the SG group across all time points. PTH levels significantly fell from 5.7 (IQR 4.2–7.4) to 5.00 (4.1–6.5) pmol/L by 24 months following SG. However, PTH levels following RYBP remained stable at 24 months, from 6.1 (IQR 4.7–8.7) to 6.4 (4.9–8.1) pmol/L. Furthermore, we failed to notice a significant improvement in PTH levels following RYBP among those with higher PTH levels preoperatively.

**Conclusion:**

Higher PTH levels following RYBP, compared to SG, may imply we are undertreating patients who are inherently subjected to a greater degree of malabsorption and underlying nutritional deficiencies. This finding calls for a tailored supplementation protocol, particularly for those with high preoperative PTH levels undergoing RYBP, to prevent deficiencies.

**Supplementary Information:**

The online version contains supplementary material available at 10.1186/s12893-021-01348-3.

## Background

Beyond lifestyle and pharmacological treatment options, bariatric surgery has become one of the most common therapeutic interventions for treating obesity. Due to the nature of the surgery, patients can develop significant postoperative micronutrient and vitamin deficiencies [1]. Of interest, calcium and vitamin D deficiencies could lead to secondary hyperparathyroidism and metabolic bone disease. As these surgeries are being performed on younger and broader cohorts, it is pertinent to elucidate these outcomes. This observational study evaluates baseline, and medium-term serum corrected calcium, vitamin D and parathyroid hormone levels following Roux-en-Y Gastric Bypass (RYBP) and Sleeve Gastrectomy (SG).

## Method

A retrospective analysis was conducted on prospectively collected data of 370 participants from the ages 17 to 71 who underwent RYBP or SG between January 2015 and December 2016 inclusive, with at least 2 years follow-up. All patients were assessed and prepared for surgery by a single multidisciplinary team consisting of a surgeon, endocrinologist, dietician, psychologist, and clinical nurse educator within a single-centre group practice in Melbourne, Australia. Preparation included education around the importance of nutritional supplementation guided by the specialist dietitian. Patients underwent either RYBP or SG depending on appropriate indications performed by two surgeons with a standardised technique. The same single-centre team delivered postoperative care and follow-up. Follow-up appointments were routinely scheduled at 3, 6, 12, 24 months and yearly after that. Further appointments were offered as required by individual circumstance as determined by the treating professional.

Laboratory results were collected preoperatively and subsequently at 6, 12 and 24 months postoperatively. Collected data includes demographic parameters, body mass index (BMI) and laboratory values (serum 25-OH vitamin D, corrected serum calcium levels, estimated glomerular filtration rate (eGFR), Haemoglobin A1C (HbA1c) and parathyroid hormone (PTH) levels. Our laboratory’s normal reference ranges are: 25-OH vitamin D > 50 nmol/L, corrected serum calcium 2.15–2.65 mmol/L, PTH 1.5–7 pmol/L. Follow-up rates were estimated as per adherence to vitamin D laboratory tests counts. Compliance to supplementation was recorded as ‘poor’ and ‘full’ following discussions with the patient by the specialist dietician or treating surgeon.

During the follow-up period, participants underwent a standard calcium and vitamin D supplementation protocol as described here. All bariatric patients were recommended 3000–5000 IU of vitamin D per day and 600–900 mg of calcium citrate per day. Further 600–900 mg of calcium through dietary intake was encouraged. Throughout the follow-up period, if vitamin D levels remained over 75 nmol/L, patients were advised to continue 1000 IU of vitamin D per day. For vitamin D levels between 50 and 70 nmol/L, patients were advised a further 2000–3000 IU per day. For vitamin D levels between 30 and 50 nmol/L, patients were advised a further 3000–5000 IU per day. If vitamin D levels dropped below 30 nmol/L, patients were given 5000 IU per day oral supplementation or a 600,000 IU intramuscular injection. Patients who were deficient before surgery were given appropriate supplementation.

Pregnant patients were excluded due to inconsistent weights and physiological fluctuations. Primary and revision bariatric surgeries were collected and noted separately to account for heterogeneous medical and nutrition status. However, statistical analyses were simplified into either RYBP or SG.

### Operative technique

Sleeve gastrectomy was performed over a 36 Fr Bougie and included antral resection after complete mobilisation of the stomach from gastro-oesophageal junction to the pylorus. Any hiatal hernia evident was repaired.

RYGB was performed, creating a gastric pouch of approximately 6 cm × 2 cm, incorporating complete exclusion of the fundus. The Biliopancreatic limb was routinely 100 cm, and the alimentary limb 90–100 cm. The anastomotic technique was standardised between the two surgeons, with the gastroenterostomy being 2/0 vicryl handsewn over a 36 Fr bougie and the entero-enterostomy being a side-to-side stapled anastomosis completed with 2/0 vicryl handsewn closure of enterotomies.

### Statistical analysis

Statistical analyses were performed with SPSS 26 (SPSS Inc Chicago). A value of p < 0.05 was considered statistically significant. As per Shapiro–Wilk criteria, normally distributed data are reported as mean ± standard deviation. Nonparametric data are reported as median (interquartile range). Student’s *t*-tests, with equal variances assumed, are used to compare the difference of normally distributed data (calcium levels) between the two surgical procedures. Likewise, Mann–Whitney tests are used to compare nonparametric variables (i.e., vitamin D and PTH levels). Preoperative data were compared with postoperative data using repeat measures ANOVA for normally distributed data and Wilcoxon-signed rank test for nonparametric data. Multivariable linear regression models were undertaken with key preoperative and postoperative variables to account for confounding variables. Common co-variates included were age, gender, BMI, eGFR, type of surgery and appropriate baseline measures. The patient count number over the same period may vary; the lowest number count was always taken to minimise errors.

## Results

We performed a total of 370 bariatric procedures, including 281 (76%) SG and 89 (24%) RYBP. The average age of the patients was 46.2 ± 0.7 years and 52.4 ± 1.1 years in the SG and RYBP group, respectively. The majority of patients were female; 75% and 87% within the SG and RYBP group, respectively. We noted no significant differences in weight loss (p = 0.845), renal function (p = 0.431) or glycaemic control (p = 0.809) between the two surgical procedures. Average preoperative BMI was 41.7 kg and 41.2 kg within the SG and RYBP cohorts, respectively.

Table 1 (see Additional file 1): Outcome measures following RYBP and SG at Baseline and 6, 12, 24 and 36 months postoperatively.

**Table 1:** Patient demographics

Preoperative data	SG	RYBP	p	
Age	46.2 ± 0.7	52.4 ± 1.1	0.43	
Gender	Male	69 (25%)	12 (14%)	0.03*
	Female	212 (75%)	77 (87%)	
Total of 370 patients	281 (100%)	89 (100%)		

Roux-en-Y Gastric Bypass (RYBP) and Sleeve Gastrectomy (SG). Mean age (± SE) in years. Gender (Proportion % within each surgical procedure). ^*^ significant at *p* < 0.05

### Serum calcium levels: RYBP and SG (Fig. 1)

**Fig. 1:**
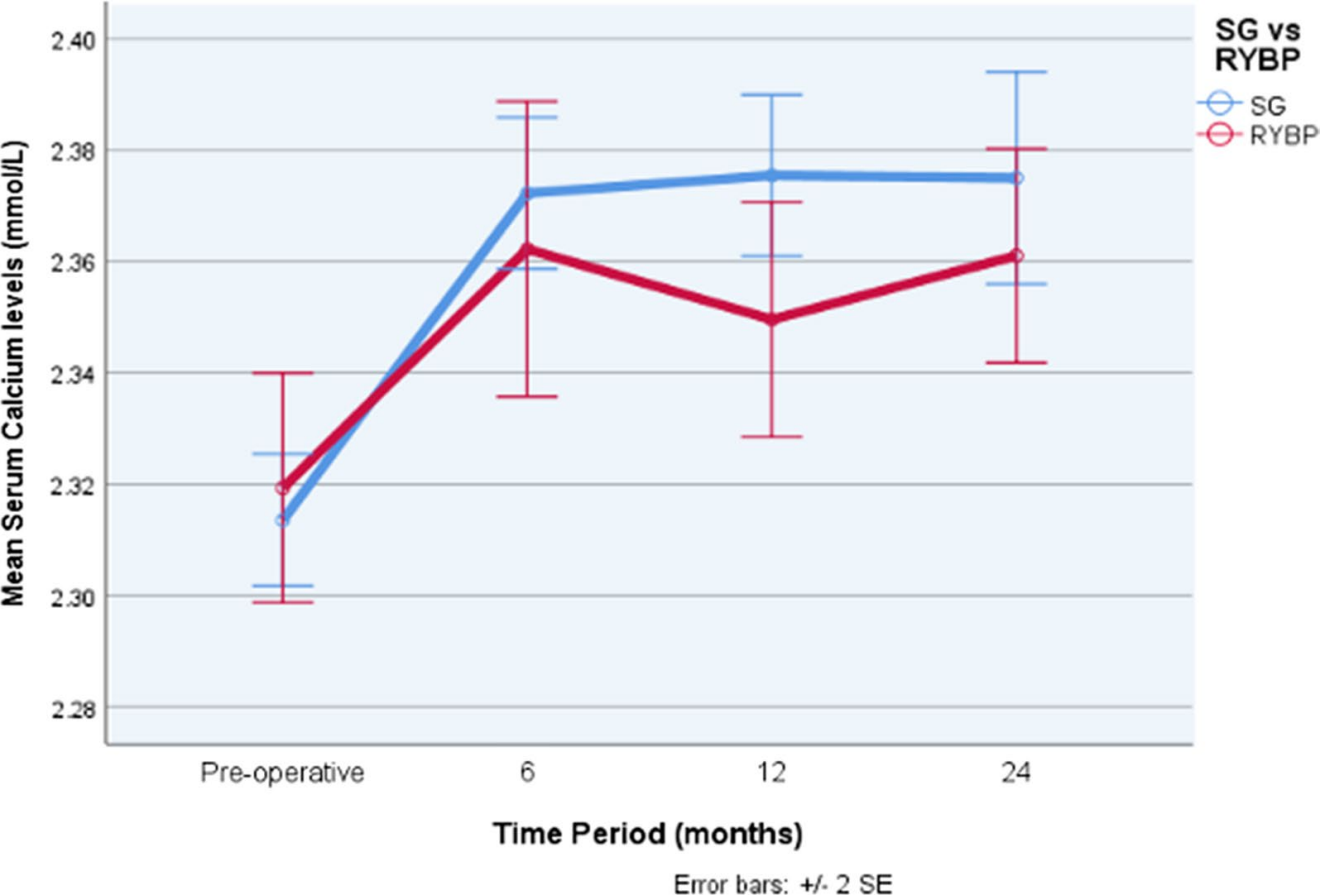
Serum corrected calcium levels following obesity surgery. Blue: sleeve gastrectomy. Red: Roux-en-Y Gastric bypass. Normal laboratory values: corrected calcium 2.15–2.65 mmol/L (shaded light blue). Error bars: ± 2 × standard error

Preoperative mean serum corrected calcium levels were 2.31 ± 0.01 mmol/L and 2.32 ± 0.01 mmol/L within the SG and RYBP cohorts, respectively. By 24 months postoperatively, serum calcium levels rose to 2.37 ± 0.10 mmol/L and 2.36 ± 0.07 mmol/L after SG and RYBP, respectively (Fig. 1). Compared to preoperative levels, calcium levels at 24 months were significantly higher following RYBP (p = 0.013) and SG (p = 0.001). There were no statistical differences in calcium levels between the two procedures across all time points (Table 1).

### Serum vitamin D levels: RYBP and SG (Fig. 2)

**Fig. 2:**
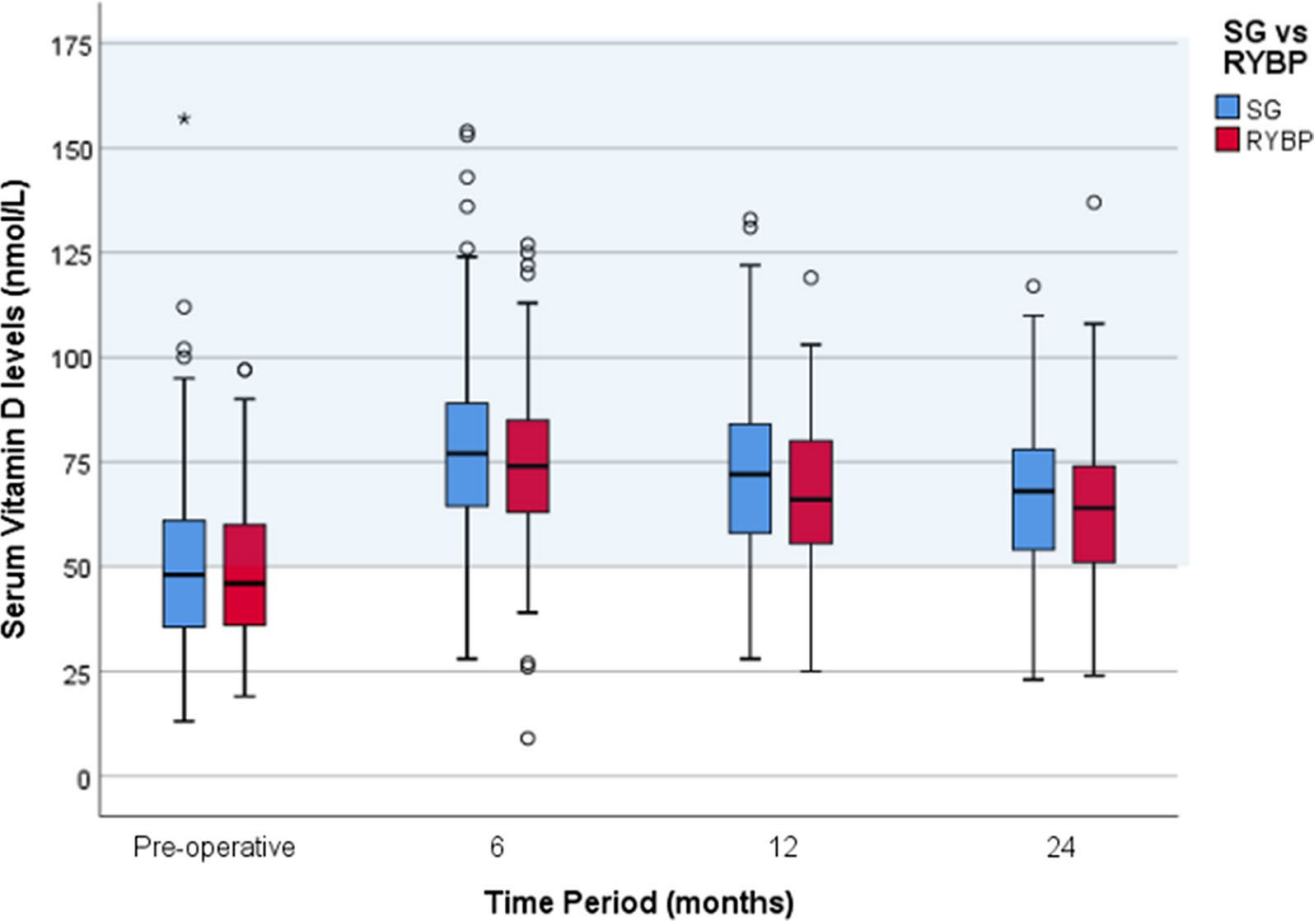
Serum vitamin D levels following obesity surgery. Blue: sleeve gastrectomy. Red: Roux-en-Y Gastric bypass. Acceptable laboratory values: 25-OH vitamin D > 50 nmol/L (shaded light blue). Outliers: circle–1.5–3 × interquartile range, asterisk– > 3 × Interquartile range above the third quartile or below the first quartile

Median preoperative vitamin D levels were 48 (IQR 35–61) nmol/L and 46 (IQR 36–60) nmol/L within the SG and RYBP cohorts, respectively. Vitamin D levels following RYBP and SG were significantly greater than preoperative levels across all follow-up time points (p < 0.01 at 6, 12 and 24 months). Vitamin D levels peaked at 6 months; 77 (IQR 64–89) nmol/L following SG and 74 (IQR 63–85) nmol/L following RYBP. Following the peak at 6 months, vitamin D levels had a significant fall by 24 months to 68 (IQR 54–79, p = 0.001) following SG and 64 (IQR 51–75, p = 0.005) following RYBP. Nonetheless, these levels at 24 months were significantly higher than those preoperatively (p = 001). (Fig. 2). Across all follow-up periods, there appear to be no significant differences in vitamin D levels between the two surgical groups.

### Parathyroid hormone (Fig. 3)

**Fig. 3:**
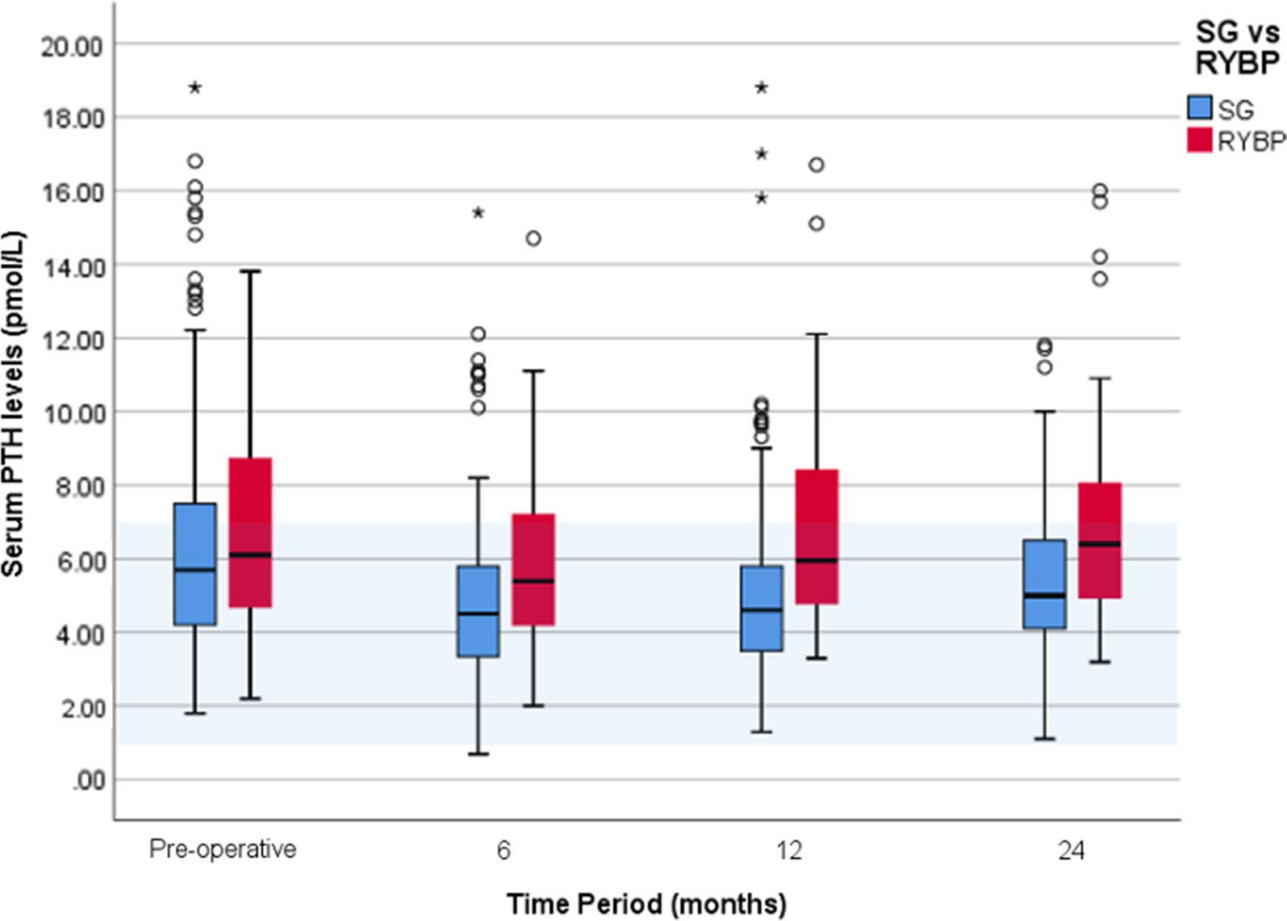
Serum parathyroid hormone levels following obesity surgery. Blue: sleeve gastrectomy. Red: Roux-en-Y Gastric bypass. Normal laboratory values: PTH 1.5-7 pmol/L (shaded blue). Outliers: circle–1.5–3 × interquartile range, asterisk– > 3 × interquartile range above the third quartile or below the first quartile

One of the most pertinent findings throughout the study period is that those who had undergone RYBP had significantly higher serum PTH levels than their SG counterparts (p < 0.01) consistently across all time points, including preoperative (Table 1). The difference in PTH levels between the two groups remains true even when adjusted for age, gender, baseline BMI, preoperative vitamin D and preoperative PTH levels.

Preoperative parathyroid hormone levels were 5.7 (IQR 4.2–7.5) pmol/L and 6.1 (IQR 4.7–8.7) pmol/L within the SG and RYBP groups, respectively. Postoperatively, these values significantly dropped to 4.5 (IQR 3.3–5.8) pmol/L and 5.4 (IQR 4.2–7.2) pmol/L at 6 months (p < 0.001), respectively (Fig. 3). By 24 months, PTH levels within the SG group remained significantly lower than preoperative levels at 5.00 (IQR 4.1–6.5) pmol/L, p < 0.05. However, by 24 months, PTH levels within the RYBP group rose back to preoperative levels 6.4 (IQR 4.9–8.1), p = 0.459.

It appears that by 24 months, those with high PTH levels (> 7.0 pmol/L) preoperatively improve following SG (from 8.9 (IQR 7.7–11.6) pmol/L to 6.55 IQR (5.3–9.23) pmol/L, p < 0.05). However, this significant improvement was not observed following RYBP as they remain at high PTH levels at 24 months; (from 8.9 IQR (8.2–9.23) pmol/L to 8.20 IQR (5.90–10.05) pmol/L, p = 0.399). Nonetheless, amongst those with high PTH levels, vitamin D levels increased significantly to acceptable levels (> 50 nmol/L) by 24 months following both surgeries (SG: from 37 IQR (28–51.5) to 64.5 IQR (54–78) nmol/L p < 0.05 and RYBP: from 42 IQR (30–59.5) nmol/L to 60 IQR (49.3–69.5) nmol/L p = 0.21). Preoperatively, the mean age of those with high PTH levels were 48.2 ± 1.1 years, whereas those with normal PTH levels (1.5–7 pmol/L) were 46.9 ± 0.8 years (t (359) = 0.97, p = 0.33) (see Additional file 2).

#### Compliance

Follow-up rates were 71%, 61% and 47%, at 6, 12 and 24 months, respectively. Of the 174 participants reviewed at 24 months, compliance to vitamin and mineral supplementation was recorded in 152 cases. 84% of these reported participants were fully compliant with taking supplementation (127 of 152 cases).

## Discussion

This study comprises one of the largest cohorts to date, evaluating the impact of commonly performed bariatric surgeries on bone metabolism over a medium-term follow-up period of 24 months.

### Serum calcium levels: RYBP and SG

This study not only investigates differences in serum calcium levels over time but also the difference between RYBP and SG. Following both surgeries, mean serum calcium levels significantly rise compared to baseline measurements by 24 months. This improvement may be due to the robust mineral supplementation protocol and the transient rise in vitamin D levels seen postoperatively. Nonetheless, these values remain within normal homeostatic levels (2.15–2.65 mmol/L) [2]. We find no significant differences in calcium levels between the two procedures.

### Serum vitamin D levels: RYBP and SG

Obesity appears to be associated with vitamin D deficiency [3]. Many observational studies have illustrated an inverse relationship between body mass index (BMI) and vitamin D status [4]. The exact pathogenesis is not clear. Mechanisms proposed include inadequate food and supplement intake, lower sunlight exposure due to lack of physical activity/body image insecurities, increased metabolic clearance and volumetric dilution vitamins (i.e. vitamin D) in the expanded fat mass [4, 5]. We did not measure the effect of solar UVB exposure or dietary intake. However, a large cohort from a single centre would experience a similar solar exposure; geographically and culturally. All patients were counsel by the same dietitian and advised according to set a protocol, with no drastic changes to a balanced diet.

This study finds that serum vitamin D levels tend to transiently rise around 6 months and subsequently drop to a steady-state level by 24 months that is greater than baseline measurements following both surgical procedures. This transient rise in vitamin D levels at 6 months is reflected in several other studies [6, 7]. A plausible explanation for this may be due to the release of sequestered vitamin D from adipose tissue with weight loss. This release may temporarily overcome the impairment in vitamin D absorption postoperatively. However, there appear to be no significant differences in vitamin D levels between the two cohorts.

The improvement in vitamin D status we observe following RYBP is contrary to many studies. A hallmark paper by Johnson et al. (2006) found a fall in vitamin D levels following RYBP [8]. This is one of the few studies on this matter with follow-up data over 12 months and well into 72 months. As vitamin D levels tend to fluctuate immediately following surgery, perhaps we may require more extended follow-up periods for these changes to become apparent.

Our findings following SG are similar to other studies which have found improvements in vitamin D levels [3, 4, 9–11]. The majority of these studies have short follow-up periods of less than 12 months. Nonetheless, we have found that this improvement is maintained even after 24 months of follow-up.

### Serum parathyroid hormone levels: RYBP and SG

In response to low calcium levels, serum parathyroid hormone levels rise to increase serum calcium—through bone resorption, increased calcitriol production and calcium reabsorption from the kidney. For this reason, PTH may be a more significant overall indicator of bone health.

Given the clinical significance of PTH, our results are surprising and may be clinically relevant. We find that PTH levels are significantly higher within the RYBP group when compared to the SG group across all time points, including baseline. However, these differences increase with time and may have more significant implications for long-term bone health. It is essential to consider the significance of the preoperative differences in PTH levels between the two cohorts. This difference may be due to disparities in co-morbidities or medications before surgery. We did not document proton-pump inhibitor (PPI) use in our patients; however, a common indication of RYGB over SG is the presence of reflux requiring PPI use. PPI’s are known to inhibit calcium absorption [12] thereby encouraging increased PTH. Many studies postulate that PTH levels rise with age and may explain our findings within the RYBP group [13]. In our study, those with high PTH levels (> 7 pmol/L) were on average 1.3 years older than those with normal PTH levels (1.5–7 pmol/L). However, we have little to no evidence to show that age is significantly greater in those with high PTH levels when compared to those with normal PTH levels in our study (p = 0.33).

Following a transient fall in PTH levels at 6 months after RYBP, these levels rise back to preoperative levels by 24 months. This fall in PTH levels may be due to the heavy supplementation protocol employed and the rise in vitamin D levels among our patients. Most studies also find higher PTH levels following RYBP [3, 4, 14–19] but not all [20–22]. Studies with stable or lower PTH post-RYBP often has low follow-up periods (< 12 months) or more robust supplementation. Despite a similar fall in PTH levels following SG at 6 months, these levels continue to remain below preoperative concentrations. Studies regarding PTH levels following SG are sparse and inconclusive, ranging from no effect [10, 23, 24] to elevated PTH levels [4, 25, 26].

We found that those with high PTH levels (greater than 7 pmol/L) preoperatively, significantly improved to normal levels by 24 months following SG but not RYBP. Nonetheless, with the standard supplement protocol employed here, vitamin D levels among this subgroup improved significantly following both surgeries by 24 months. Perhaps, the improvements in vitamin D levels seen in both high-risk groups may be attributed to the excellent compliance to supplementation of 84% we have observed at 24 months. However, the lack of significant improvement seen in PTH levels in the RYBP group with high preoperative levels, from 8.9 to 8.2 pmol/L, is concerning. This may be attributable to the duodenal bypass in RYGP, which circumvents most of the active calcium reabsorption seen in the duodenum and upper jejunum [27]. Perhaps we are undertreating these high-risk patients, despite their excellent compliance with supplementation, which is evident in their vitamin D levels (from 42 to 60 nmol/L). Perhaps higher PTH levels are required following RYBP to maintain the adequate calcium and vitamin D levels we have observed. RYBP may lead to a more malabsorptive state than SG and would ultimately require a tailored supplementation protocol.

## Limitations

Significant limitations in our study include the influence of incomplete data in medical records and losses to follow up. These limitations may overestimate the strength of the results as it may be the most compliant patients who attend follow-up meetings at 24 months. Compliance with nutritional supplementation proved challenging to identify; however, it strongly influences the outcomes measured. Despite these limitations, this is one of the few studies that compare SG and RYBP with a large sample size over a medium-term follow-up period. Additionally, the frequency of use of proton-pump inhibitors within the two surgical groups may have assisted in discerning the differences seen in outcomes.

It appears that the nutritional protocol from this single-centre group practice was able to overcome nutritional deficiency among the measured outcomes. There continues to be a lack of consensus regarding adequate supplementation to optimise postoperative nutritional requirements. A 2013 update from the American Association of Clinical endocrinologists (AACE), the American Society for Metabolic and Bariatric Surgery (ASMBS) and The Obesity Society (TOS) recommend an initial dosage of 3000 IU vitamin D (titrated to therapeutic levels) and 1200–1500 mg of calcium for RYBP and SG patients [28]. There are no studies that discriminate the differences in nutritional requirements between different bariatric surgeries. As per guidelines, both RYBP and SG receive the same supplementation dosage. Despite apparent physiological differences between the two different surgeries, supplementation does not reflect this [28]. Future research should incorporate the influence of nutritional supplementation for different bariatric procedures.

## Future studies

Further studies examining long-term postoperative outcomes regarding bone health and its relationship to appropriate supplementation would be most helpful. Using serum calcium and PTH as a marker of overall calcium status is unreliable as it is subject to several physiological feedback mechanisms. Gletsu-miller et al. points out that bone is a significant storage reservoir for calcium [29]. Although a costly alternative, measuring bone density via Dual-ray absorptiometry may yield a more robust understanding of the significance of vitamin D, PTH and calcium levels post-surgery [29].

## Conclusions

With adequate supplementation, postoperative mean serum calcium and vitamin D levels tend to significantly rise but remain within the normal homeostatic range, with no statistical difference between Roux-en-Y-Gastric bypass and Sleeve Gastrectomy. Serum parathyroid hormones levels are statistically different between the two surgical procedures across all time points. Higher parathyroid hormone levels following Roux-en-Y Gastric Bypass may be of significance to poor long-term bone health and the need for a more robust micronutrient supplementation protocol.

## Supplementary Information

**Additional file 1**. Outcome measures following Roux-en-Y Gastric Bypass (RYBP) and Sleeve gastrectomy (SG) at baseline, 6, 12 and 24 postoperatively.

**Additional file 2.** Distribution of age within those with high PTH levels (> 7 pmol/L, green) and those with normal PTH levels (1.5–7 pmol/L, gray).

## Data Availability

The datasets used and/or analysed during the current study are available from the corresponding author on reasonable request.

## Abbreviations

RYBP: Roux-en-Y Gastric Bypass
SG: Sleeve gastrectomy
25-OH Vitamin D: 25-Hydroxy-vitamin D
Vit D: Vitamin D
VDD: Vitamin D deficient
VDI: Vitamin D insufficient
PTH: Parathyroid Hormone
eGFR: Estimated Glomerular Filtration Rate
Hb1Ac: Haemoglobin A1c
BMI: Body Mass Index (kg/m^2^)
AACE: American Association of Clinical Endocrinologists
ASMBS: The American Society for Metabolic and Bariatric Surgery
TOS: The Obesity Society
