# Supplementary material for: Preventing calcium and vitamin D deficiencies following weight loss and metabolic surgery
Source: BMC Surg. 2021 Sep 25;21:351. doi: 10.1186/s12893-021-01348-3 (PMC8464128; doi:10.1186/s12893-021-01348-3)
Supplement: Supplementary file 1 — Additional file 1. Outcome measures following Roux-en-Y Gastric Bypass (RYBP) and Sleeve gastrectomy (SG) at baseline, 6, 12 and 24 postoperatively. [file 12893_2021_1348_MOESM1_ESM.docx]

Additional File 1: Outcome measures following Roux-en-Y Gastric Bypass (RYBP) and Sleeve Gastrectomy (SG) at baseline, 6, 12 and 24 postoperatively. Body mass index (BMI). Parathyroid Hormone (PTH).

*p<0.05 significant. Data represented as mean ± SD, median (IQR), count (proportion %)

|  | Preoperative Data | | | 6 months | | | 12 months | | | 24 Months | | |
| --- | --- | --- | --- | --- | --- | --- | --- | --- | --- | --- | --- | --- |
|  | SG | RYBP | p | SG | RYBP | p | SG | RYBP | p | SG | RYBP | p |
| BMI (kg/m^2^) | 41.7  (37.8 -47.9) | 41.2 (35.8 -47.3) | 0.462 | 32.8 (29.3 – 38.1) | 33.6  (28.7 -39.3) | 0.886 | 30.8 (27.1-35.1) | 30.5 (27.3- 38.1) | 0.949 | 31.4 (27.1 – 34.8) | 30.4 (27.0 -36.5) | 0.817 |
| Count | 281 | 88 |  | 231 | 74 |  | 156 | 64 |  | 85 | 37 |  |
| Calcium levels  (mmol/L) | 2.31 ± 0.10 | 2.32 ± 0.10 | 0.628 | 2.37 ± 0.09 | 2.36 ± 0.11 | 0.472 | 2.37 ± 0.09 | 2.35 ± 0.08 | 0.052 | 2.37 ± 0.10 | 2.36 ± 0.07 | 0.380 |
| Count | 278 | 89 |  | 190 | 65 |  | 157 | 63 |  | 119 | 51 |  |
| Vitamin D levels  (nmol/L) | 48  (35-61) | 46  (36-60) | 0.975 | 77  (64-89) | 74  (63-85) | 0.432 | 73  (58-84) | 66  (55-80) | 0.187 | 68  (54-79) | 64  (51-75) | 0.175 |
| Count | 279 | 87 |  | 199 | 65 |  | 162 | 63 |  | 122 | 53 |  |
| PTH levels (pmol/L) | 5.7  (4.2-7.5) | 6.1  (4.7-8.7) | 0.018* | 4.5 (3.3- 5.8) | 5.4  (4.2- 7.2) | 0.001* | 4.60 (3.5-5.9) | 5.95 (4.8- 8.4) | 0.000* | 5.00 (4.1- 6.5) | 6.40 (4.9- 8.1) | 0.000* |
| Count | 274 | 87 |  | 192 | 62 |  | 149 | 62 |  | 117 | 51 |  |
