# Supplementary material for: Preventing calcium and vitamin D deficiencies following weight loss and metabolic surgery
Source: BMC Surg. 2021 Sep 25;21:351. doi: 10.1186/s12893-021-01348-3 (PMC8464128; doi:10.1186/s12893-021-01348-3)

Additional File 2: Distribution of age within those with high PTH levels (>7pmol/L, green) and those with normal PTH levels (1.5 – 7 pmol/L, gray).


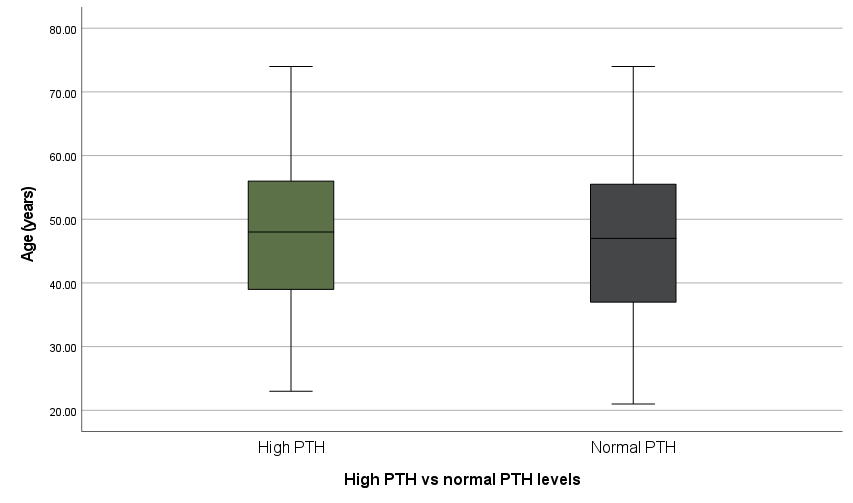

Supplement: Supplementary file 2 — Additional file 2. Distribution of age within those with high PTH levels (> 7 pmol/L, green) and those with normal PTH levels (1.5–7 pmol/L, gray). [file 12893_2021_1348_MOESM2_ESM.docx]
